# Simplified Rules for Serviceability Control of FRPRC Elements

**DOI:** 10.3390/polym14122513

**Published:** 2022-06-20

**Authors:** Tomislav Kišiček, Tvrtko Renić, Ivan Hafner, Mislav Stepinac

**Affiliations:** Faculty of Civil Engineering, University of Zagreb, 10000 Zagreb, Croatia; tomislav.kisicek@grad.unizg.hr (T.K.); ivan.hafner@grad.unizg.hr (I.H.); mislav.stepinac@grad.unizg.hr (M.S.)

**Keywords:** FRP reinforcement, serviceability limit states, design charts

## Abstract

Serviceability limit states are very important in the design of reinforced concrete elements but they are complex to calculate. Simplified serviceability calculations are provided in EN 1992-1-1 (2013) for steel reinforced elements. The crack widths are assumed to be acceptable if the bar diameters or bar spacings are not too large, while deflections are acceptable if the slenderness is not too large. In recent decades, FRP bars have become an adequate replacement for steel bars, especially in aggressive environments. The calculation procedures for FRP-reinforced concrete elements (FRPRC) were developed from calculation methods for steel reinforced elements. The first part of this paper demonstrates the procedures and parametric investigation for calculating the maximum bar diameter and bar spacing for the purpose of controlling the crack width, focusing on calculations for the maximum bar diameter for which cracks widths are acceptable. The second part of the paper demonstrates the procedures and parametric calculations for the slenderness limits for concrete elements reinforced with FRP bars in order to satisfy the usual deflection limits. Due to the different modulus of elasticity values of FRP and steel, the tables used for steel cannot be used for concrete beams reinforced with FRP bars. Therefore, new tables and diagrams are proposed in the paper. The new tables and diagrams for the maximum allowable bar diameters for the different modulus of elasticity values of FRP can be useful for the rapid control of the crack width in FRPRC elements. They are conservative compared to the exact calculations because some assumptions taken in the calculations are different to those taken in the exact calculation procedure for the crack width. The results of parametric calculations for the slenderness limits for FRPRC elements are provided in the form of a diagram for different concrete classes. Satisfying the slenderness from these curves will result in a smaller deflection than that allowed for each parameter related to that class of concrete.

## 1. Introduction

In addition to satisfying the load bearing capacity of reinforced concrete elements, satisfying serviceability requirements is also important. In case of elements reinforced with FRP bars (FRPRC), satisfying serviceability limit state (SLS) requirements could be a problem (ACI 440 [1], fib Bulletin 40 [2], Barris et al. [3,4], CNR-DT-203 [5]). Usually, deflection control and crack width control are checked. Detailed checks can be complex. Simplifications can help during conceptual phase of the design, but also in the design of simple structures. To simplify the calculations for the crack widths for elements subjected to bending and reinforced with steel reinforcement, the EN 1992-1-1 [6] proposes a bar diameter (table 7.2(N)) or bar spacing (table 7.3(N)) that conforms to an allowable crack width (Mosley et al. [7], Zilch and Zehetmeier [8], Bhatt et al. [9] and Dahlgreen et al. [10]). For cracking induced by loads, the crack width will be smaller than the maximum allowable value if the conditions either from table 7.2(N) or table 7.3(N) are satisfied. If restrained imposed deformations cause the cracking of the element, satisfying the conditions from table 7.2(N) is enough [5], (Narayanan et al. [11]). In numerous studies, equations for the calculation of the bar spacing or allowable bar diameter limits are provided. Corres Peiretti et al. [12] provided procedures in accordance with EN 1992-1-1 [6]. Eurocode 2—Commentary [13] provided similar explanations.

In addition to the crack width, it is also important to satisfy the deflection limits of reinforced concrete elements. To simplify the calculations, EN 1992-1-1 [6] provides allowable slenderness limits (table 7.4(N) and Equation (7.16)) for elements reinforced with steel reinforcement. If the slenderness of a reinforced concrete element is smaller than the limit value, it can be assumed that the deflection of such elements will be within allowable limits. These slenderness limits were calculated by a parametric study described in Corres Peiretti et al. [14] and Eurocode 2—Commentary [13]. The analysis was based on a simply supported beam or slab. Many parameters that influence the slenderness limit were considered, such as complex time history (the time of load application according to the type of load, the influence of relative humidity (creep and shrinkage effect), the amount and distribution of reinforcement, the concrete class, and the proportion of the different types of loads in the total load).

In recent decades, FRP reinforcement has become a good replacement for steel reinforcement, especially in aggressive environments ([1,2], Zoghi [15]). Calculation methods for reinforced concrete elements with FRP bars were developed from calculation methods for reinforced concrete elements with steel bars ([1,2,3,4,5], Pilakoutas et al. [16], Pecce et al. [17]). Furthermore, in fib Bulletin 40 [2], it is claimed that there are no fundamental reasons why the principles behind the verification of SLS for FRPRC elements should differ from those already established in the codes of practice for steel RC elements. However, the actual limits could be different to account for the differences in both short and long-term properties between steel and FRP reinforcement. Also, the limits on deflections for steel RC elements are equally applicable to FRPRC elements. However, the ratios of effective span to depth (*L*/*d*) are not. ACI 440 [1] considered that these ratios are not conservative for FRPRC and recommended further studies. More and more, these rules are implemented in national codes, and they will be implemented in the new set of Eurocodes.

Tables 7.2(N), 7.3(N) and 7.4(N), which convey slenderness limits from [6] used for steel, should not be used in calculations for FRPRC elements because the modulus of elasticity of FRP and steel are different. Consequently, this paper describes the parametric procedures for FRP bars and proposes new tables and diagrams. The calculations were made according to rules from [6], paragraph 7.3.4, Equations (7.8) and (7.9) and paragraph 7.4, but the properties of FRP bars were used instead of those of steel material.

## 2. Theoretical Background of Bar Diameter and Spacing Limits

In accordance with EN 1992-1-1 (2013) [6], the calculation of the design crack width can be carried out using the equation:

(1) $$w_{k}=s_{r,\max}\cdot (\varepsilon_{\mathrm{sm}}-\varepsilon_{\mathrm{cm}})$$

where *s*_r,max_ is the largest expected crack spacing and (*ε*_sm_ − *ε*_cm_) is the difference of mean reinforcement strain and mean concrete strain at the reinforcement level between two cracks. The largest expected crack spacing, in the case of the spacing between the main reinforcement being smaller than 5(c + φ/2), is:

(2) $$s_{r,\max}=k_{3}\cdot c+k_{1}\cdot k_{2}\cdot k_{4}\cdot \phi /\rho_{p,\mathrm{eff}}$$

Concrete cover, *c*, is taken here as a typical value of *c* = 25 mm and φ is the bar diameter. The coefficients *k*_1_ to *k*_4_ are defined as: *k*_1_ = 0.8 (for high bond bars), *k*_2_ = 0.5 (for bending), *k*_3_ = 3.4 and *k*_4_ = 0.425. Barris [3] suggests that the same value of *k*_1_ can be used for FRP bars. The values of coefficients *k*_2_ to *k*_4_ are independent of the reinforcement material.

The equations for the largest bar spacing and diameter that satisfy the maximum allowable crack width are determined using Equations (1) and (2) and following assumptions.

The minimum value of the reinforcement ratio, *ρ*_p,eff_, is determined from the following equation:

(3) $$\rho_{p,\mathrm{eff}}=\frac{A_{s}}{A_{c,\mathrm{eff}}}=\frac{k_{c}\cdot f_{\mathrm{ct},\mathrm{eff}}\cdot \frac{A_{\mathrm{ct}}}{\sigma_{s}}}{2.5\cdot b\cdot (h-d)}$$

In order to avoid failure when the first crack appears, the reinforcement area, *A*_s_, is required. This assumption is conservative, as shown in following paragraphs. The value *f*_ct,eff_ is the mean concrete tensile strength that is effective when the cracks are first expected to appear. Area *A*_ct_ = 0.5·*b*·*h* is the tensile cross-section area before the first crack appears. The value *A*_c,eff_ is an effective cracked cross-section tensile area. If *d* = 0.9·*h* and *k_c_* = 0.4 (used for bending) are assumed, then the reinforcement ratio, *ρ*_p,eff_, is:

(4) $$\rho_{p,\mathrm{eff}}=\frac{0.8\cdot f_{\mathrm{ct},\mathrm{eff}}}{\sigma_{s}}$$

The maximum expected crack spacing equation can be rearranged as:

(5) $$s_{r,\max}=3.4\cdot 25+0.8\cdot 0.5\cdot 0.425\cdot \phi /\rho_{p,\mathrm{eff}}=85+0.2125\cdot \phi \cdot \frac{\sigma_{s}}{f_{\mathrm{ct},\mathrm{eff}}}$$

The difference of mean reinforcement strain and mean concrete strain between two cracks, according to [12], is:

(6) $$\varepsilon_{\mathrm{sm}}-\varepsilon_{\mathrm{cm}}=\frac{\sigma_{s}}{E_{s}}\cdot \left(1-k_{t}\cdot \left(\frac{\sigma_{\mathrm{sr}}}{\sigma_{s}}\right)\right)$$

This is the same equation as shown in [6]. The stress in the reinforcement caused by the cracking moment can be derived from Equation (3):

(7) $$\sigma_{\mathrm{sr}}=\frac{k_{c}\cdot f_{\mathrm{ct},\mathrm{eff}}\cdot h}{5\cdot (h-d)\cdot \rho_{p,\mathrm{eff}}}$$

Taking account of Equations (7) and (4) and assumptions *d* = 0.9·*h*, *k_t_* = 0.4 (used for long term loading) and *k_c_* = 0.4 (used for bending), Equation (6) may be written as:

(8) $$\varepsilon_{\mathrm{sm}}-\varepsilon_{\mathrm{cm}}=\frac{\sigma_{s}}{E_{s}}-0.32\cdot \frac{f_{\mathrm{ct},\mathrm{eff}}}{0.8\cdot \frac{f_{\mathrm{ct},\mathrm{eff}}}{\sigma_{s}}\cdot E_{s}}=\frac{\sigma_{s}}{E_{s}}-0.32\cdot \frac{\sigma_{s}}{0.8\cdot E_{s}}=\frac{\sigma_{s}}{E_{s}}-0.4\cdot \frac{\sigma_{s}}{E_{s}}=0.6\cdot \frac{\sigma_{s}}{E_{s}}$$

Using the values from Equations (5) and (8) in Equation (1), the bar diameter limit value is:

(9) $$\phi =\frac{f_{\mathrm{ct},\mathrm{eff}}\cdot E_{s}\cdot w_{k}-51\cdot \sigma_{s}\cdot f_{\mathrm{ct},\mathrm{eff}}}{0.1275\cdot \sigma_{s}{}^{2}}$$

The following equation connects the bar spacing and the bar diameter:

(10) $$\rho_{p,\mathrm{eff}}=\frac{A_{s}}{A_{c,\mathrm{eff}}}=\frac{\frac{\phi^{2}\cdot \pi }{4}\cdot \frac{b}{s_{b,\max}}}{b\cdot 2.5\cdot (h-d)}$$

Using the assumptions from Equation (4) and *d* = 0.9·*h*, Equation (10) can be rewritten as:

(11) $$s_{b,\max}=\frac{\phi^{2}\cdot \pi }{\rho_{p,\mathrm{eff}}\cdot h}=\frac{\phi^{2}\cdot \pi \cdot \sigma_{s}}{0.8\cdot f_{\mathrm{ct},\mathrm{eff}}\cdot h}$$

It is apparent from Equation (11) that the allowable bar spacing is dependent on the maximum allowable bar diameter, the stress in the reinforcement, previous assumptions and on the cross-section height. According to background documents and [6], it is not clear which cross-section height and bar diameter were used to obtain the values shown in table 7.3(N). When bending of the elements occurs either the bar diameter or bar spacing need to be satisfied. Since the bar spacing is dependent on several parameters, only the diameter of the bar will be considered in further analysis.

**Figure 1:**
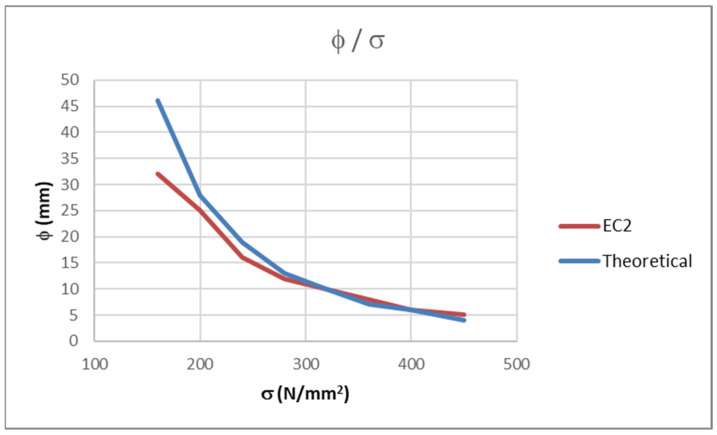
Comparison of values from [6] and theoretical values (Equation (9)).

The values obtained using Equation (9) are slightly different to those from table 7.2(N) [6]. The values provided in the table in [6] are usually smaller than the ones that are obtained by Equation (9) because the values of the bar diameters given in [6] are rounded down to match the available bar diameters in the industry. A comparison between the values from [6] and from Equation (9) is presented in Table 1 and in Figure 1.

Since the assumptions *d* = 0.9·*h*, *k*_c_ = 0.4, *f*_ct,eff_ = 2.9 N/mm^2^ and that the element is subjected to bending are used, the values in table 7.2(N) should be multiplied with the coefficients shown in Equations (12) and (13) in the case of using other values:

For a partially compressed cross-section:

(12) $$\frac{f_{\mathrm{ct},\mathrm{eff}}}{2.9}\cdot \frac{k_{c}\cdot h_{\mathrm{cr}}}{2\cdot (h-d)}$$

For a cross-section in tension:

(13) $$\frac{f_{\mathrm{ct},\mathrm{eff}}}{2.9}\cdot \frac{k_{c}\cdot h_{\mathrm{cr}}}{4\cdot (h-d)}$$

## 3. Crack Width Control of FRPRC Elements

When corrosion is expected to be a problem, such as in a maritime environment, using FRP bars instead of steel has become standard for reinforced concrete elements. In addition to the fact that FRP reinforcement is resistant to corrosion, it has many other positive qualities such as a low weight, a high tensile strength, a small relaxation, good behavior under dynamic loadings and moisture resistance. Also, it is an electric isolator and magnetically neutral. When comparing FRP with steel, it has some deficiencies, such as its non-ductile behavior, creep rupture, lower modulus of elasticity values and a lower coefficient of thermal expansion. Finally, the bending of cured FRP bars can damage them and they are sensitive to ultraviolet exposure [1,2,3,4,5,15,16,17,18].

The calculation procedures for FRPRC elements are the same as those for steel-reinforced elements. Designers should keep in mind that FRP bars are known for their non-ductile behavior [1,2,3,4,5,15,16,17,18]. The design of the ULS of steel reinforced elements often relies on the ductility of bars. The same procedures for crack width control can be used for both materials since FRP and steel bars remain elastic at the SLS. Also, in fib Bulletin 40 [2], it is claimed that crack width limits are larger for FRPRC elements than for elements reinforced with steel. However, these limits may not be adequate for structures exposed to extreme and aggressive environmental conditions or for those designed to be watertight. In the absence of more information, limitations suggested for steel RC structures can be adopted for FRPRC structures.

Tables 7.2(N) and 7.3(N), from [6], which are used for steel reinforced elements, cannot be used in calculations for FRPRC elements due to FRP having a different modulus of elasticity values to that of steel. This is because the modulus of elasticity values for FRP bars can vary from 30 to 47 GPa for BFRP (basalt), 45 to 60 GPa for GFRP (glass) and 130 to 165 GPa for CFRP (carbon) bars. These values have been taken from various manufacturer brochures for FRP bars. For each considered modulus of elasticity, a maximum allowable bar diameter can be determined depending on the stress in the reinforcement.

Usually, for steel-reinforced concrete elements, a stress of 310 MPa is assumed. It is from that assumption that the allowable bar diameter is calculated. This is an acceptable assumption for steel-reinforced elements because most of the steel used has the same yield strength of 500 MPa and because the ultimate limit state (ULS) often governs the design. The limit of 310 MPa is derived from that. For FRPRC, it is rarely the case that the ULS governs the design. The range of strengths used for FRP bars are usually much wider, and because of that, calculating stresses in FRP bars from an assumed diameter is more convenient. Therefore, Equation (9) can be rearranged as so:

(14) $$0.1275\cdot \phi \cdot \sigma_{f}{}^{2}+51\cdot f_{\mathrm{ct},\mathrm{eff}}\cdot \sigma_{f}-f_{\mathrm{ct},\mathrm{eff}}\cdot E_{f}\cdot w_{k}=0$$

Using Equation (14), for the same maximum diameters of steel bars, from table 7.2(N) [6], stresses in FRP bars can be determined for each modulus of elasticity of FRP bars (30, 60, 130 and 165 GPa).

**Figure 2:**
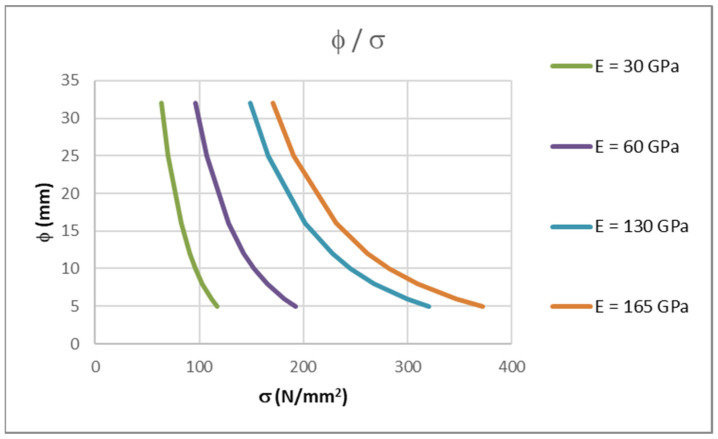
Maximum allowable bar diameter to stress in bar ratios for different FRP modulus of elasticity values and for *k*_1_ = 0.8.

The results are shown in Table 2 and in Figure 2. As it can be seen, for the same bar diameter, values for the allowable stress in the reinforcement increase with an increase in the modulus of elasticity of FRP. For a specific bar diameter, stresses change approximately linearly with the change in modulus of elasticity. Therefore, for values between those in this paper, linear interpolation can be used.

It is important to state that these curves were determined for concrete class C30/37 and all of the aforementioned assumptions that are equal to those for steel reinforcement. If different parameters are used, the maximum allowable bar diameters from these curves can be corrected with Equations (12) and (13). In these equations, the tensile strength of the concrete, height, effective depth of the cross-section and loading conditions are considered.

If the idea is to determine stresses in bars with different assumptions in terms of concrete cover, *c*, the bond coefficient, *k*_1_ and a different crack width, *w*_k_, Equation (14) can be expressed in a more general way:

(15) $$0.159375\cdot k_{1}\cdot \phi \cdot \sigma_{f}{}^{2}+2.04\cdot c\cdot f_{\mathrm{ct},\mathrm{eff}}\cdot \sigma_{f}-f_{\mathrm{ct},\mathrm{eff}}\cdot E_{f}\cdot w_{k}=0$$

**Figure 3:**
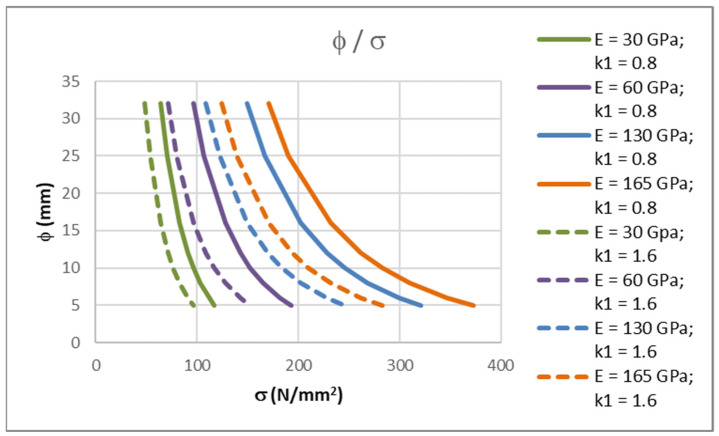
Comparison of maximum allowable bar diameter to stress in bar ratios for different FRP modulus of elasticity values and for *k*_1_ = 0.8 and *k*_1_ = 1.6.

One of the most questionable parameters is the bond coefficient for FRP bars. According to [2], the bond coefficient, *k*_1_, should be taken as 0.8 for high bond bars (as in the case of steel reinforcement) or 1.6 for plain bars, and, according to [4], it should be taken as 1.6. These two values can be interpreted as the lower and higher boundaries for the bond properties of FRP bars. Since FRP bars can have many different surface geometries, the bond properties should further be experimentally determined. In Table 3, the results of the calculation according to Equation (15) for plain bars (*k*_1_ = 1.6) are shown. To compare the differences between the stresses in FRP reinforcement for high bond and plain bars, the results from Table 2 and Table 3 are shown in Figure 3.

It is clear that the influence of the bond coefficient is not small. The difference in terms of the results becomes more pronounced as the modulus of elasticity value of the FRP bars increase. In the absence of further investigations and values for the bond coefficient, linear interpolation between lower and higher values can be used.

In a previous analysis and in [6,12], the concrete cover was taken as *c* = 25 mm. Barris et al. [4] and Caldentey et al. [19] investigated the influence of the concrete cover on the crack width. It was concluded that the concrete cover significantly influences the crack width. Greater values of concrete cover result in a greater value in terms of the crack width.

**Figure 4:**
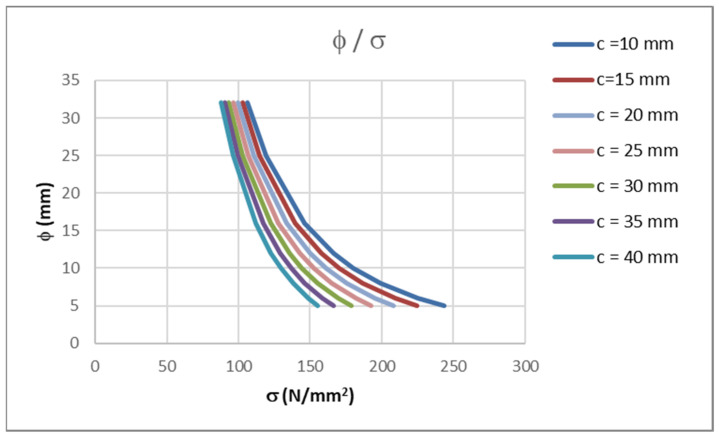
Stresses in FRP bars for maximum allowable bar diameter and different concrete covers.

Table 4 and Figure 4 show the results using Equation (15) for FRP bars with a modulus of *E*_f_ = 60 GPa and values of concrete cover that are between 10 and 40 mm. Choosing the concrete cover value of 25 mm appears to be suitable choice for slab and beam elements of standard dimensions. If a more detailed calculation is required (e.g., if an element is supposed to be watertight), Equation (15) can be used. For standard uses, where the crack width is controlled only for aesthetic reasons, earlier values are appropriate.

## 4. Parametric Analysis of Maximum Bar Diameters

Visual Basic for Applications (VBA) procedures were used because crack width calculations are complex. In addition to the crack width calculation, the deflection control and load bearing capacity procedures were developed. In most cases, the ULS does not govern the design of FRPRC elements. In most cases, the reinforcement required for the ULS does not satisfy the SLS [1,2,3,4,5]. The main reason for this phenomenon is that FRP usually has a lower modulus of elasticity compared to steel. Therefore, both SLS and ULS conditions should be considered in the design of FRPRC elements.

**Figure 5:**
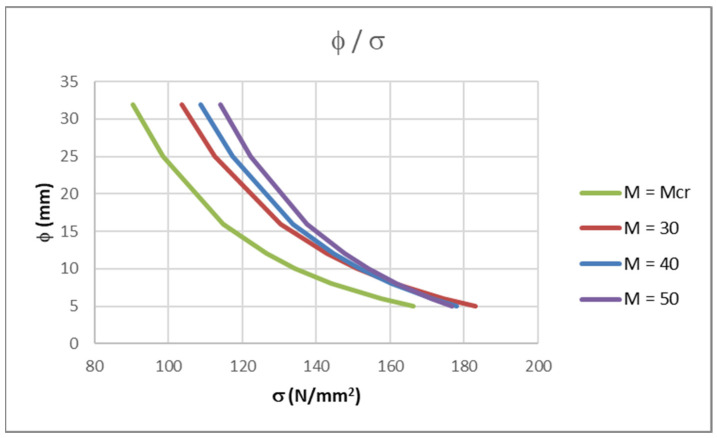
Results of parametric analysis.

A parametric analysis was conducted to verify Equations (1)–(9) and Equations (14) and (15) for the calculation of the maximum allowable bar diameters and confirmed that using them ensures that the crack width is smaller than *w*_k_ = 0.3 mm. The aim was to determine the stresses in different diameter bars for different bending moments while the parameter of a maximum crack width of *w*_k_ = 0.3 mm was satisfied. The cross-section used was a rectangular slab of 100 cm width, 20 cm height, using 25 mm concrete cover, *c*, and a concrete class C30/37. The modulus of elasticity of the FRP bars that were used was *E*_f_ = 60 GPa. The calculation from table 7.2(N) in [6] was conducted for bar diameters. First, a bending moment that causes the cracking of the cross-section, equal to *M*_cr_ = 19.33 kNm, was considered. Three other values were considered next: *M*_Ed_ = 30 kNm (1.5·*M*_cr_), *M*_Ed_ = 40 kNm (2.0·*M*_cr_) and *M*_Ed_ = 50 kNm (2.5 *M*_cr_). The tensile reinforcement area in the cross-section was determined to ensure a characteristic crack width of *w*_k_ = 0.3 mm for each bending moment. The results are presented in Table 5 and in Figure 5.

As the bending moment increases, the curves φ/σ shift to the right. This means that if the maximum allowable bar diameter for the curve *M* = *M*_cr_ is used, the calculation of the crack width will be conservative. However, for the same degree of reinforcement stress, if the bending moment is increased, the maximum allowable bar diameter will also increase. To conclude, choosing a lower bar diameter for a certain amount of stress than that of *M* = *M*_cr_ results in the crack width requirements being satisfied. This is in accordance with [6,12,13].

When considering an adequate modulus of elasticity for FRP bars, the stresses in bars for a bending moment of *M* = *M*_cr_, as determined by this parametric analysis, should be the same as those shown in Table 2. Using the same input data as in the analysis above, the calculation of the stresses in FRP bars, for *M* = *M*_cr_ and for each bar diameter and each modulus of elasticity of FRP (from Table 2), was conducted. The calculation was carried out using the same VBA procedure mentioned above for determining crack width. Table 6 contains the results.

**Figure 6:**
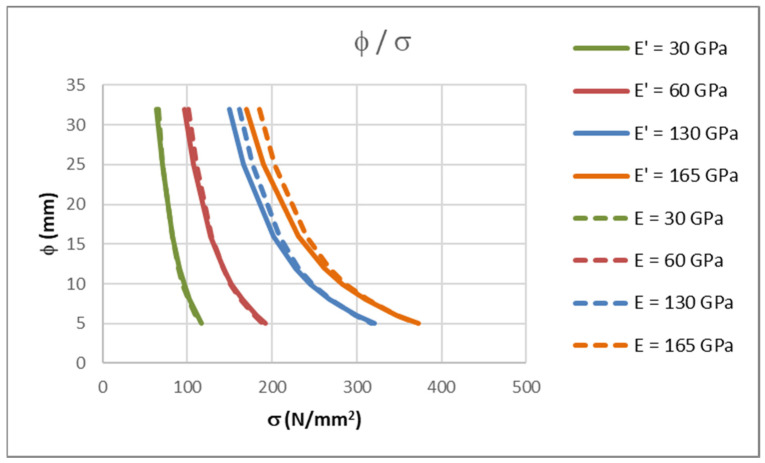
Stresses in reinforcement for maximum allowable bar diameter calculated using the VBA procedure for crack width control.

The results from Table 2 and Table 6 are compared in Figure 6. FRP bars with a modulus of elasticity of 30, 60, 130 and 165 GPa were considered.

In Figure 6, the curves marked with E’ were calculated using the VBA procedure, and curves marked with an E were calculated by using the simple procedure from the first two chapters. The corresponding curves have a little discrepancy between them if the same modulus of elasticity is considered. With higher modulus of elasticity values and larger bar diameters, the differences are greater. The values from Table 2 are conservative, which justifies their usage.

Differences arise with regard to the maximum allowable bar diameter for crack width control due to the assumptions considered during the calculations of parameters in tables and diagrams. 

The calculations in first two chapters assume the effective depth of the cross-section *d* = 0.9·*h*, while the calculations in this chapter assume an effective depth *d* = *h* − (*c* + φ/2). Therefore, if the concrete cover is fixed and the bar diameter is variable, then the *d*/*h* ratio is also variable. Furthermore, the stress in the reinforcement is calculated using a lever arm *z* = 0.8·*h*, while calculations in this chapter use a lever arm calculated from the geometrical properties of the cracked cross-section. Finally, the effective height of concrete in tension is assumed to be *h*_eff_ = 2.5 (*h* − *d*). This is not always the case. In the parametric analysis from this chapter, the effective height of concrete in tension is taken as (*h* − *x*)/3, which is the smaller value. All these differences lead to discrepancies in results that explain why values from Table 2 are conservative. It is important to note that this analysis was calculated for a cross-section height *h* = 20 cm, which is a typical floor slab height. If a thicker slab was used, the results would be different, especially because of the method used to calculate the effective depth in the exact calculation.

## 5. Slenderness Limits According to Procedure Used for Steel Reinforcement

In addition to satisfying the ULS and crack width of reinforced concrete members, it is important to satisfy allowable deflections. Unfortunately, deflection calculations are complicated and the related procedures are long and involve many parameters that impact the final result.

To simplify the calculation, it is possible to determine the slenderness of the observed element for which the deflection would be smaller than the allowable limit.

Recently, several authors dealt with that problem and provided the formulations for the slenderness of FRP-reinforced concrete elements to satisfy the deflection limits (Ospina and Gross [20] and Veysey and Bischoff [21]). Additionally, the new calculation procedures for satisfying the SLS were provided in [3].

EN 1992-1-1 [6] in table 7.4(N) provides the slenderness limits for elements reinforced with steel reinforcement. These limits can be expressed in the form of a reinforcement ratio-span/effective depth ratio curves (*ρ* – *L*/*d*). The procedure for deriving such curves for elements with steel reinforcement has been described by Corres Peiretti et al. [14] and Eurocode 2—Commentary [13].

Therefore, the basic idea in this paper was to check if it is possible to use reinforcement ratio-span/effective depth ratio curves (*ρ* – *L*/*d*), according to [14], that would render the deflection calculation unnecessary for FRP-reinforced concrete beams.

In this paper, a parametric analysis of slenderness limits like the one described for steel reinforcement [14] was carried out, but, instead of steel reinforcement, FRP reinforcement was used in the analysis. The procedure for steel reinforcement was first used to assess its applicability with respect to FRPRC beams. Although the logic should be the same, since FRPRC is usually governed by the SLS, the procedure used for steel might need to be modified. This was tested and is commented on in the following paragraphs of this section.

The analysis was based on a simply supported beam. The first step of the parametric analysis is to choose a cross-section (*b*/*h*) with an effective height of *d* = 0.9·*h*. Next, a rectangular cross-section is chosen which is 100 cm wide and 30 cm high. In addition to the dimensions of the cross-section, the concrete class of C30/37 is chosen.

The reinforcement ratio range is chosen from *ρ* = 0.001–0.045. For each reinforcement ratio, the following procedure is repeated:

1. For each reinforcement ratio *ρ*, the area of tensile FRP reinforcement is calculated.
2. Based on the FRP reinforcement area and the dimensions of the cross-section, the ultimate bending moment (*M*_ULS_) is calculated.
3. In this step, the span of the beam is presumed. The total load is calculated from the ultimate bending moment and beam span. It is assumed that the load is uniformly distributed.

According to [13,14], the percentage of self-weight (*g*_1_), additional dead load (flooring and partitions) (*g*_2_) and live load (*q*) concerning the total load is taken according to Spanish practice (for one-way slab) as 45, 30 and 25%, respectively. Furthermore, the live load is taken with the coefficient for a quasi-permanent action of *ψ*_02_ = 0.3, which is typical of residential and office areas.

4. From the previous estimation of the dead to live load ratio, the load for serviceability limit state is calculated.
5. From the span presumed in step 3 and loads from step 4, the deflection calculation can be carried out. The complex load history is also considered. The self-weight load, *g*_1_, is applied at *t_g_*_1_ = 10 days and the remaining dead load, *g*_2_, is applied at *t_g_*_2_ = 60 days. Finally, the quasi-permanent live load application, *ψ*_2_*q*, is set at *t*_q_ = 365 days.

The deflection is calculated according to the procedure based on [6] considering the load history.

6. The procedure is iteratively repeated from step 3 while the total deflection is not equal to the maximum allowable deflection of *L*/250. If that condition is satisfied, the value *L*/*d* is calculated for the chosen reinforcement ratio from step 1.

**Figure 7:**
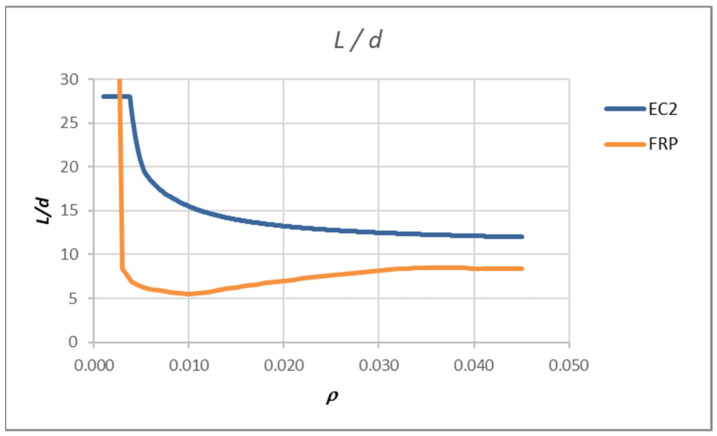
Results of analysis-limit span to depth ratio for FRPRC element compared with one for element with steel reinforcement according to [13,14].

The values *L*/*d*, which are calculated by that procedure, are lower than those for steel reinforcement and their usage would lead to very thick cross-sections reinforced with FRP bars. The reason for this lies in steps 2–4 of the procedure. For steel RC, it makes sense to determine the amount of reinforcement from the ULS loads, but for FRPRC, this reinforcement is too small. Figure 7 shows the span/effective depth ratio curves (*ρ* – *L*/*d*) which were calculated for a rectangular slab cross-section of 100 cm width and 30 cm height, using 25 mm of concrete cover, *c*, and a concrete class of C30/37. The modulus of elasticity of the used FRP bars was *E*_f_ = 50 GPa. The curve denoted as FRP was compared with the curve according to EN 1992-1-1 [6]. For example, if reinforcement ratio of 0.005 and a slab span of 200 cm are assumed, the *L*/*d* ratio that satisfies the deflection is 6.40. According to Figure 7, the thickness of the slab reinforced with FRP should be 200/6.40 = 31.35 cm.

This clearly shows that the procedure used for steel RC should be modified. The proposed modifications are presented in the following paragraphs.

## 6. Parametric Analysis of Slenderness Limits for Elements Reinforced with Steel or FRP According to Theoretical Procedure

In order to obtain more realistic values for the *L*/*d* ratio, a parametric analysis of single span, simply supported slabs was carried out. The calculations were made using a procedure made in VBA according to [6] (Kišiček et al. [22,23] and Renić et al. [24]). Slab thicknesses between 17.5 cm and 30 cm were considered, and spans varied between 4.0 and 8.0 m. The additional dead load was taken as 2.0 kN/m^2^ and the live load, for residential buildings, was taken as 2.0 kN/m^2^. For comparison, the analysis was made for elements with steel and FRP reinforcement. The considered mechanical properties of GFRP bars were: *E*_f_ = 50 GPa, *ε*_fu_ = 0.025. C30/37 concrete and B500B steel were considered in this analysis.

First, the ULS was calculated to obtain the required tensile reinforcement. The crack width was checked using the reinforcement required for satisfying the ULS. If the crack width was not satisfied, the amount of reinforcement required to satisfy the crack width was determined and provided. When the reinforcement satisfied both the ULS and crack width, the deflections were finally checked. Also, if the deflection was not satisfied, the amount of reinforcement required to satisfy the deflection was determined.

The results of the analysis for slabs reinforced with steel reinforcement are provided in Table 7. For all the elements with a span *L* = 4.0 m, there were no cracks because the bending moments for the ULS were smaller than *M*_cr_ (the cracking moment of the cross-section). Also, the deflections were smaller than *L*/250. In such cases, the ULS governs the design.

For the elements with a span of *L* = 5.0 m and thicknesses between 17.5 and 19 cm, the limit state of cracking was satisfied while deflections were not. Even with the increase in the area of reinforcement, it was not possible to satisfy the deflection of *L*/250. For cross-sections with thicknesses between 19.5 and 23 cm, limit state of cracking could be satisfied by a slight increase in the reinforcement area, which was also the case for satisfying the deflections. The exception was the slab with a thickness of 23 cm, where the deflection was satisfied with the same area of reinforcement, as it was required for satisfying the crack width. For the thicker slabs, those up to 30 cm, there was no appearance of cracks and the deflections satisfies the *L*/250 limit.

For the elements with a span of *L* = 6.0 m and thicknesses between 17.5 and 24 cm, the limit state of cracking was satisfied using the reinforcement that satisfies the ULS. For the elements with thickness between 17.5 and 23.5 cm, even with the increase in the area of reinforcement, it was not possible to satisfy the deflection of *L*/250; however, if thickness were 24 cm, increasing of reinforcement lead to a deflection of *L*/250. For cross-sections with thicknesses between 24.5 and 30 cm, the limit state of cracking could be satisfied with a slight increase in the reinforcement area, which was also the case for satisfying the deflections for cross-sections with thicknessed between 24.5 and 29 cm. Slabs with thicknesses of 29.5 and 30 cm had deflections of less than *L*/250 with the same area of reinforcement, as it was required for satisfying the crack width.

For the elements with a span of *L* = 7.0 m and thicknesses between 17.5 and 29 cm, the limit state of cracking was satisfied. For the elements with thicknesses between 17.5 and 27.5 cm, even with the increase in the area of reinforcement, it was not possible to satisfy the deflection of *L*/250. For cross-sections with thicknesses between 29.5 and 30 cm, the limit state of cracking could be satisfied with a slight increase in the reinforcement area. Satisfying the deflections for cross-sections with thicknesses between 28 and 30 cm was possible with a significant increase in the area of reinforcement.

For all the elements with a span *L* = 8.0 m, the limit state of cracking was satisfied, but even with the increase in the area of reinforcement, it was not possible to satisfy the deflection of *L*/250.

The results of analysis for slabs reinforced with GFRP reinforcement are provided in Table 8. For all the elements with a span of *L* = 4.0 m, there were no cracks because the bending moments for the ULS were smaller than *M*_cr_ (the cracking moment of the cross-section). Also, the deflections were smaller than *L*/250. In such cases, the ULS governs the design.

For the elements with a span of *L* = 5.0 m and thicknesses between 17.5 and 23 cm, the limit state of cracking was satisfied with a significant increase in the reinforcement area (around five times the area required to satisfy the ULS). For the thicker slabs, those up to 30 cm, no cracks appeared. For slabs with thicknesses between 17.5 and 19 cm, even with the increase in the area of reinforcement, it was not possible to satisfy the deflection of *L*/250. Up to thicknesses of 23 cm, this was possible, but the amount of reinforcement could be substantial, causing congestion issues. For slabs with thicknesses from 23.5 and 30 cm, the deflections were satisfied for the ULS reinforcement area.

For all the elements with a span of *L* = 6.0 m, the limit state of cracking was satisfied with a significant increase in the reinforcement area (around five times the area required to satisfy the ULS). For slabs with thicknesses between 17.5 and 23.5 cm, even with the increase in the area of reinforcement, it was not possible to satisfy the deflection of *L*/250. For cross-sections with thicknesses between 24 and 30 cm, the deflections could be satisfied with an increase in reinforcement area, but the amount of reinforcement could be substantial, causing congestion issues.

For all the elements with a span of *L* = 7.0 m, the limit state of cracking was satisfied with a significant increase in the reinforcement area (two to five times of the area for the ULS). For the elements with thicknesses between 17.5 and 27.5 cm, even with the increase in the area of reinforcement, it was not possible to satisfy the deflection of *L*/250. Satisfying the deflections for cross-sections with thicknesses between 28 and 30 cm was possible with a significant increase in the area of reinforcement, but the amount of reinforcement was substantial, causing congestion issues.

For all the elements with a span of *L* = 8.0 m, the limit state of cracking was satisfied, but even with the increase in the area of reinforcement, it was not possible to satisfy the deflection of *L*/250.

The results obtained showed that the most demanding limit to satisfy is the deflection limit. In some cases, even if the ULS was satisfied, increasing the amount of reinforcement did not lead to satisfying the deflections. This can be seen in final columns of Table 7 and Table 8, where the reinforcement ratio has large values.

The results show that the SLS is not always satisfied for the same amount of reinforcement which satisfies the ULS. In many cases, the amount of reinforcement which satisfied the ULS was not enough for crack control. If the crack width is satisfied with the increase in the area of reinforcement, it is not a guarantee that the deflections will be. In many cases, increasing the area of reinforcement did not lead to satisfying the deflections. The only way to satisfy deflections is to increase the thickness of the cross-section. A basic assumption in the analysis undertaken in [13,14] was that both the SLS and ULS are satisfied with the same amount of reinforcement. That assumption (as shown in Section 5) led to very low values in terms of the slenderness limits for elements reinforced with FRP reinforcement if the same procedure was used.

## 7. Parametric Analysis of Slenderness Limits for FRPRC Elements

In order to obtain more realistic values in terms of slenderness limits, *L*/*d*, for FRPRC elements, a new parametric analysis was carried out. It was based on a simply supported slab with a cross-section 100 cm wide and 30 cm high, with a concrete cover of *c* = 25 mm, for which the reinforcement ratio varied from a minimum to a maximum value, i.e., between 0.0013 and 0.02. For each reinforcement ratio, the element was loaded with the design loads for the SLS, from 5.0 to 15.0 kN/m^2^. These amounts appeared to be reasonable for typical slabs used in residential and office buildings. For a 12 cm thick slab (thinner plates may not provide a diaphragm action), with an additional dead load of 2.0 kN/m^2^ and moving load of 2.0 kN/m^2^, q_SLS_ = 5.6 kN/m^2^. For a 30 cm thick slab, with an additional dead load of 2.5 kN/m^2^ and moving load of 4.0 kN/m^2^, q_SLS_ = 11.2 kN/m^2^.

The goal was to calculate a span of the element for which the resulting deflection would be equal to *L*/250. This resulted in curves depending on various other parameters: concrete class, FRP reinforcement modulus of elasticity and cross-section height. It must be noted that the amount of reinforcement which satisfies the deflection for certain amount of load does not imply that the ULS and crack limit are satisfied. The ultimate limit state and crack limit must be checked separately.

**Figure 8:**
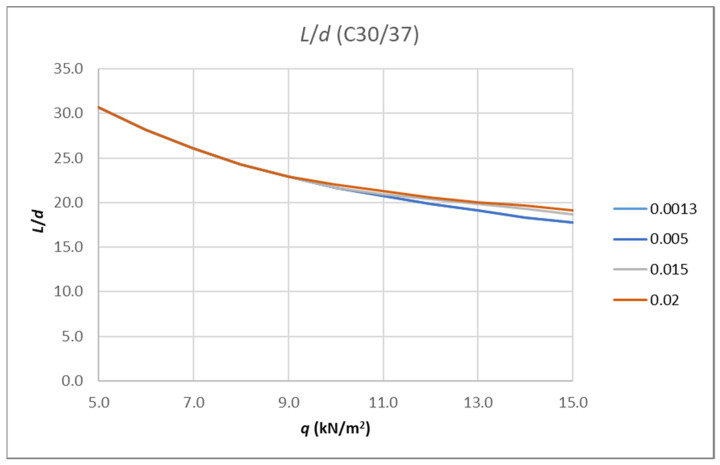
Results of analysis-limit span to depth ratio depending on amount of load for fixed value of concrete class and different reinforcement ratio.

There are many curves representing the results depending on the input parameters. Figure 8 shows the results for the aforementioned cross-section made from C30/37 concrete reinforced with FRP bars with a modulus of elasticity of *E*_f_ = 60 GPa. There are four curves for the following reinforcement ratios: 0.0013, 0.005, 0.015 and 0.02.

**Figure 9:**
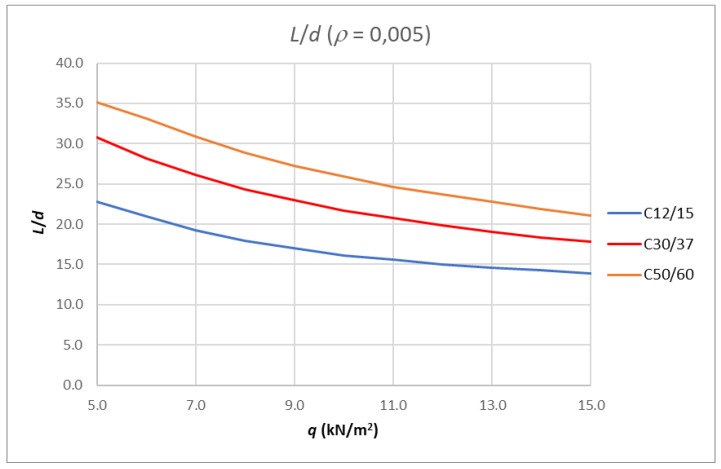
Results of analysis-limit span to depth ratio depending on amount of load for fixed reinforcement ratio and different concrete class.

If the fixed reinforcement ratio is taken, e.g., 0.005, and the concrete class is varied, the resulting slenderness limits *L*/*d* curves are shown in Figure 9.

**Figure 10:**
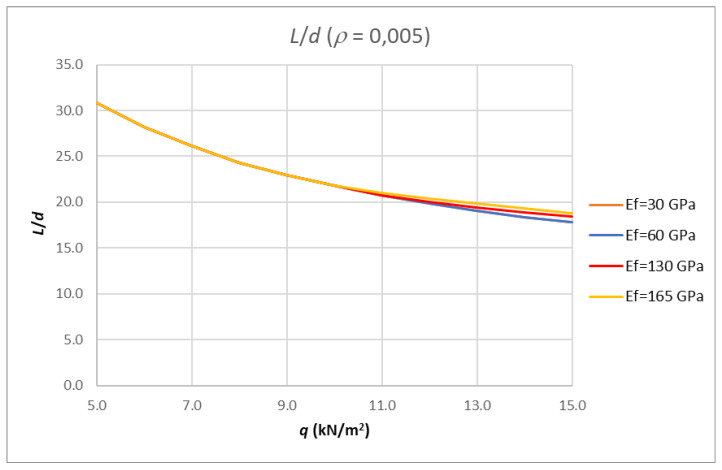
Results of analysis-limit span to depth ratio depending on amount of load for fixed reinforcement ratio and different modulus of elasticity value for FRP reinforcement.

The results for the same reinforcement ratio, a concrete class of C30/37 and different modulus of elasticity values for the FRP reinforcement are shown in Figure 10.

**Figure 11:**
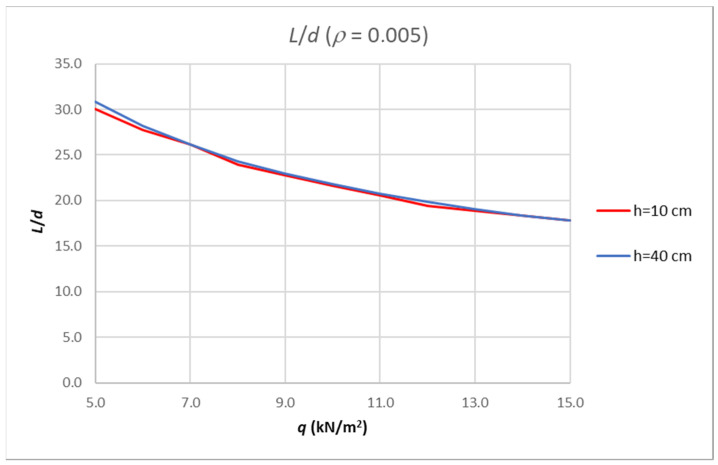
Results of analysis-limit span to depth ratio depending on amount of load for fixed reinforcement ratio and different height of cross-section.

Figure 11 represents the results if different cross-section heights are used. This is the only parameter that does not significantly affect the slenderness limit curves.

**Figure 12:**
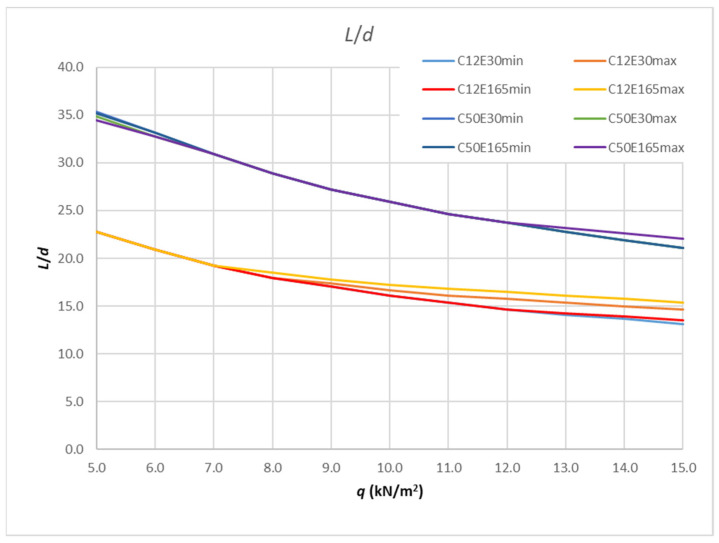
Results of parametric analysis.

Given that the values of slenderness limits are influenced by several parameters, such as the concrete class, the reinforcement ratio and the elasticity modulus of the FRP reinforcement, the results of the analysis are represented by a large number of curves that are difficult to display with one or several expressions that would take into account all these parameters. Table 9 and Figure 12 show the results for the calculation of the boundary slenderness curves for the smallest and largest class of concrete considered, i.e., C12/15 and C50/60. For each of these concrete classes, a calculation of the minimum and maximum reinforcement ratio (0.0013 and 0.02) was carried out, taking into account the smallest and the largest observed modulus of elasticity for FRP reinforcement (30 and 165 GPa). Each class of concrete provided an array of the boundary slenderness and all the possible modulus of elasticity values and reinforcement ratios.

**Figure 13:**
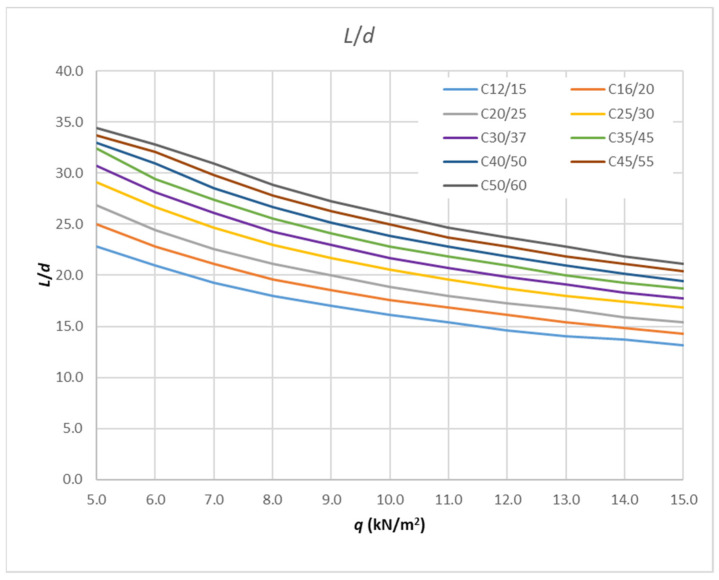
Slenderness envelope curves for each concrete class (lower bound values).

The maximum difference between the values in Table 9 for concrete C12/15 is 15% for the maximum load (when varying *E*_f_ and *ρ*), while for concrete C50/60 it is 3% for the minimum loads and 4% for the maximum load. For a load ranging from 5 to 7 kN/m^2^, there is no difference in the slenderness values for C12/15, with the same being true for a load ranging from 7 to 12 kN/m^2^ for C50/60. Finally, for each concrete class, lower boundary values are taken for slenderness limit values. Satisfying the slenderness from these curves will result in a smaller deflection than that allowed for each parameter related to that concrete class. Therefore, the result of the calculation will be on the safe side. Figure 13 provides these curves and the points values of each curve that are provided in Table 10.

## 8. Conclusions

Several conclusions can be taken as a result of this parametric research. These are:

- FRPRC elements are sensitive to the SLS, even when the ULS is satisfied. As it is stated in the literature, calculation procedures for crack width control and the deflections of steel-reinforced concrete elements can be used for FRPRC elements. This is possible because steel and FRP reinforcements are both in the required elastic region considering the SLS.
- Due to the different modulus of elasticity values of FRP and steel, restrictions from EN 1992-1-1 [6] relating to the bar diameter or bar spacing that satisfies an allowable crack width cannot be used in calculations for concrete beams reinforced with FRP bars.
- The new tables and diagrams for maximum allowable bar diameters for different modulus of elasticity of FRP provided could be useful for the rapid control of the crack width in FRPRC elements. These diagrams are conservative compared to the exact calculations. Some assumptions taken in the calculations for the diagrams are different to those taken in the exact calculation procedure for the crack width.
- It is also important to satisfy the deflection of reinforced concrete elements. EN 1992-1-1 [6] provides the allowable slenderness limits of such elements to simplify the calculations. These slenderness limits were calculated by the parametric study which is described in [13,14]. The values *L/d* that were calculated by that procedure in this paper for FRPRC elements are lower than those for steel reinforcement and their usage would lead to very thick cross-sections.
- To verify the obtained values, a parametric calculation of slabs of different spans and thicknesses reinforced with steel or FRP reinforcement was carried out. In many cases the amount of reinforcement which satisfied the ULS was not enough for crack control. If the crack width was satisfied by increasing the area of reinforcement, it was not guaranteed that the deflections would be. In many cases, increasing the area of reinforcement did lead to satisfying the deflections. The only way to satisfy deflections is to increase the thickness of the cross-section.
- A basic assumption in analysis undertaken in [13,14] was that both the SLS and ULS are satisfied with same amount of reinforcement. That assumption led to very low values of slenderness limits for elements reinforced with FRP reinforcement when the same procedure was used.
- It must be noted that the amount of reinforcement which satisfies the deflection for certain loads does not imply that the ULS and crack limit are satisfied. The ultimate limit state and crack limit must be checked separately.
- To obtain more realistic values for the slenderness limits, *L/d*, for FRPRC elements, a new parametric analysis was carried out. The reinforcement ratio varied between 0.0013 and 0.02. For each reinforcement ratio, the element was loaded with loads ranging from 5.0 to 15.0 kN/m^2^. The goal was to calculate a span of elements for which the resulting deflection would be equal to L/250. Each class of concrete resulted in an array of boundary slenderness values and all possible modulus of elasticity values and reinforcement ratios. Finally, for each concrete class, lower boundary values were taken for slenderness limit values. Satisfying the slenderness from these curves resulted in a smaller deflection than that allowed for each parameter related to that class of concrete.

## Figures and Tables

**Table 1:** Comparison of values from [6] and theoretical values (Equation (9)).

*σ*_s_ (N/mm^2^)	EN φ (mm)	Theoretical φ (mm)
160	32	46
200	25	28
240	16	19
280	12	13
320	10	10
360	8	7
400	6	6
450	5	4

**Table 2:** Stresses in FRP bars for maximum allowable bar diameter and *k*_1_ = 0.8.

φ (mm)	*E* = 30 (GPa) *σ*_f_ (N/mm^2^)	*E* = 60 (GPa) *σ*_f_ (N/mm^2^)	*E* = 130 (GPa) *σ*_f_ (N/mm^2^)	*E* = 165 (GPa) *σ*_f_ (N/mm^2^)
32	63.88	96.43	149.35	170.32
25	70.22	106.86	166.59	190.28
16	82.53	127.77	201.98	231.48
12	90.93	142.60	227.81	261.76
10	96.38	152.49	245.43	282.52
8	103.13	165.06	268.29	309.59
6	111.81	181.86	299.80	347.17
5	117.23	192.77	320.88	372.50

**Table 3:** Stresses in FRP bars for maximum allowable bar diameter and *k*_1_ = 1.6.

φ (mm)	*E* = 30 (GPa) *σ*_f_ (N/mm^2^)	*E* = 60 (GPa) *σ*_f_ (N/mm^2^)	*E* = 130 (GPa) *σ*_f_ (N/mm^2^)	*E* = 165 (GPa) *σ*_f_ (N/mm^2^)
32	48.21	71.43	109.02	123.88
25	53.43	79.63	122.10	138.91
16	63.88	96.43	149.35	170.32
12	71.30	108.66	169.60	193.77
10	76.24	116.98	183.59	210.03
8	82.53	127.77	201.98	231.48
6	90.93	142.60	227.81	261.76
5	96.38	152.49	245.43	282.52

**Table 4:** Stresses in FRP bars for maximum allowable bar diameter and different concrete covers.

φ (mm)	*c* = 10 (mm) *σ*_f_ (N/mm^2^)	*c* = 15 (mm) *σ*_f_ (N/mm^2^)	*c* = 20 (mm) *σ*_f_ (N/mm^2^)	*c* = 25 (mm) *σ*_f_ (N/mm^2^)	*c* = 30 (mm) *σ*_f_ (N/mm^2^)	*c* = 35 (mm) *σ*_f_ (N/mm^2^)	*c* = 40 (mm) *σ*_f_ (N/mm^2^)
32	106.09	102.76	99.54	96.43	93.43	90.55	87.77
25	119.03	114.81	110.75	106.86	103.12	99.55	96.13
16	146.12	139.69	133.57	127.77	122.27	117.07	112.15
12	166.39	157.97	150.05	142.60	135.60	129.05	122.91
10	180.46	170.51	161.19	152.49	144.38	136.82	129.81
8	199.07	186.87	175.54	165.06	155.37	146.45	138.23
6	225.40	209.58	195.09	181.86	169.82	158.86	148.91
5	243.49	224.89	208.02	192.77	179.01	166.62	155.47

**Table 5:** Results of parametric analysis.

Calculation φ (mm)	*M* = *M*_cr_ (kNm) *σ*_f_ (N/mm^2^)	*M* = 30 (kNm) *σ*_f_ (N/mm^2^)	*M* = 40 (kNm) *σ*_f_ (N/mm^2^)	*M* = 50 (kNm) *σ*_f_ (N/mm^2^)
32	101.20	116.50	123.00	129.50
25	110.30	126.80	132.80	139.00
16	128.90	147.40	152.00	157.30
12	142.40	161.90	165.00	169.40
10	151.40	171.50	173.50	177.10
8	163.00	183.50	183.90	186.50
6	178.70	199.40	197.30	198.20
5	188.90	209.50	205.60	205.40

**Table 6:** Stresses in reinforcement for maximum allowable bar diameter calculated with VBA procedure for crack width control.

φ (mm)	*E* = 30 (GPa) *σ*_f_ (N/mm^2^)	*E* = 60 (GPa) *σ*_f_ (N/mm^2^)	*E* = 130 (GPa) *σ*_f_ (N/mm^2^)	*E* = 165 (GPa) *σ*_f_ (N/mm^2^)
32	65.70	101.20	161.20	185.60
25	71.20	110.30	176.40	203.40
16	82.20	128.90	208.20	240.50
12	89.90	142.40	231.70	268.10
10	94.90	151.40	247.80	287.10
8	101.20	163.00	268.80	312.00
6	109.40	178.70	297.80	346.60
5	114.60	188.90	317.40	370.10

**Table 7:** Results of parametric analysis for steel-reinforced beams.

ULS						Cracks				Deflections						
*L* (m)	*h* (cm)	*M*_Ed_ (kNm)	*A*_f1_ (cm^2^)	*A*_f1,min_ (cm^2^)	*M*_Ed_ (kNm)	*w* (mm)	*A*_f1_ (cm^2^)	*w* (mm)	*A*_f1_ (cm^2^)	*f* (cm)	*l*/250 (cm)	*A*_f1_ (cm^2^)	*f* (cm)	*A*_f1_ (cm^2^)	*L/d*	*ρ*
4	17.5	23.21	3.81	2.19	13.95	0.00	3.81			0.66	1.6	3.81			27.59	0.0026
4	30.0	31.65	4.07	4.07	20.20	0.00	4.07			0.20	1.6	4.07			14.81	0.0015
5	17.5	36.27	6.04	2.19	21.80	0.26	6.04			4.13	2.0	6.04	2.44	9062	34.48	6.2497
5	19.0	37.85	5.68	2.41	22.97	0.29	5.68			3.46	2.0	5.68	2.08	8514	31.25	5.3215
5	19.5	38.38	5.57	2.49	23.36	0.30	5.57	0.30	5.60	3.25	2.0	5.60	2.00	29.63	30.30	0.0180
5	22.5	41.54	5.06	2.94	25.70	0.38	5.06	0.30	5.75	2.13	2.0	5.75	2.00	6.66	25.64	0.0034
5	23.0	42.07	5.00	3.02	26.09	0.40	5.00	0.30	5.78	1.98	2.0	5.78			25.00	0.0029
5	23.5	42.60	4.93	3.09	26.48	0.00	4.93			0.80	2.0	4.93			24.39	0.0024
5	30.0	49.45	4.31	4.07	31.56	0.00	4.31			0.47	2.0	4.31			18.52	0.0016
6	17.5	52.23	8.89	2.19	31.39	0.22	8.89			7.21	2.4	8.89	4.12	13,341	41.38	9.2010
6	23.5	61.34	7.18	3.09	38.14	0.29	7.18			4.15	2.4	7.18	2.41	10,764	29.27	5.2508
6	24.0	62.10	7.08	3.17	38.70	0.30	7.08			3.98	2.4	7.08	2.40	38.26	28.57	0.0182
6	24.5	62.86	6.99	3.24	39.26	0.31	6.99	0.30	7.10	3.78	2.40	7.10	2.40	27.82	27.91	0.0129
6	29.0	69.69	6.36	3.92	44.33	0.40	6.36	0.30	7.40	2.41	2.4	7.40	2.40	7.46	23.08	0.0029
6	29.5	70.45	6.31	4.00	44.89	0.41	6.31	0.30	7.43	2.29	2.4	7.43			22.64	0.0028
6	30.0	71.21	6.25	4.07	45.45	0.42	6.25	0.30	7.46	2.18	2.4	7.46			22.22	0.0028
7	17.5	71.09	12.48	2.19	42.72	0.18	12.48			11.09	2.8	12.48	6.40	18,713	48.28	12.906
7	27.5	91.76	9.00	3.69	58.03	0.29	9.00			5.07	2.8	9.00	2.85	13,497	28.57	5.5093
7	28.0	92.79	8.91	3.77	58.80	0.29	8.91			4.91	2.8	8.91	2.80	85.71	28.00	0.0343
7	29.0	94.86	8.74	3.92	60.33	0.30	8.74			4.60	2.8	8.74	2.80	40.30	26.92	0.0155
7	29.5	95.89	8.66	4.00	61.10	0.31	8.66	0.30	8.74	4.43	2.8	8.74	2.80	32.74	26.42	0.0124
7	30.0	96.93	8.58	4.07	61.86	0.31	8.58	0.30	8.78	4.26	2.8	8.78	2.80	27.65	25.93	0.0102
8	17.5	92.85	16.94	2.19	55.80	0.15	16.94			15.95	3.2	16.94	9.47	25,407	55.17	17.522
8	30.0	126.60	11.33	4.07	80.80	0.27	11.33			6.53	3.2	11.33	3.59	16,991	29.63	6.2933

**Table 8:** Results of parametric analysis for FRP-reinforced beams.

ULS						Cracks				Deflections						
*L* (m)	*h* (cm)	*M*_Ed_ (kNm)	*A*_f1_ (cm^2^)	*A*_f1,min_ (cm^2^)	*M*_Ed_ (kNm)	*w* (mm)	*A*_f1_ (cm^2^)	*w* (mm)	*A*_f1_ (cm^2^)	*f* (cm)	*l*/250 (cm)	*A*_f1_ (cm^2^)	*f* (cm)	*A*_f1_ (cm^2^)	*L/d*	*ρ*
4	17.5	23.21	1.89	1.89	13.95	0.00	1.89			0.64	1.6	1.89			27.59	0.0013
4	30.0	31.65	3.51	3.51	20.20	0.00	3.51			0.19	1.6	3.51			14.81	0.0013
5	17.5	36.27	2.42	1.89	21.80	6.31	2.42	0.30	12.95	5.92	2.0	12.95	2.44	19,429	34.48	13.3993
5	19.0	37.85	2.27	2.08	22.97	7.27	2.27	0.30	12.63	4.85	2.0	12.63	2.08	18,939	31.25	11.8369
5	19.5	38.38	2.23	2.15	23.36	7.62	2.23	0.30	12.65	4.51	2.0	12.65	2.00	130.95	30.30	0.0794
5	23.0	42.07	2.60	2.60	26.09	6.07	2.60	0.30	12.91	2.68	2.0	12.91	2.00	22.81	25.00	0.0114
5	23.5	42.60	2.67	2.67	26.48	0.00	2.67			0.78	2.0	2.67			24.39	0.0013
5	30.0	49.45	3.51	3.51	31.56	0.00	3.51			0.45	2.0	3.51			18.52	0.0013
6	17.5	52.23	4.36	1.89	31.39	3.54	4.36	0.30	17.92	10.63	2.4	17.92	4.12	26,874	41.38	18.5342
6	23.5	61.34	2.87	2.67	38.14	7.28	2.87	0.30	16.03	5.83	2.4	16.03	2.41	24,047	29.27	11.7305
6	24.0	62.10	2.83	2.73	38.70	7.55	2.83	0.30	16.05	5.54	2.4	16.05	2.40	169.18	28.57	0.0806
6	30.0	71.21	3.51	3.51	45.45	4.76	3.51	0.30	16.36	2.98	2.4	16.36	2.40	24.42	22.22	0.0090
7	17.5	71.09	9.31	1.89	42.72	1.33	9.31	0.30	23.14	16.84	2.8	23.14	6.40	34,705	48.28	23.9352
7	27.5	91.76	3.60	3.19	58.03	6.81	3.60	0.30	20.33	7.09	2.8	20.33	2.85	30,488	28.57	12.4444
7	28.0	92.79	3.56	3.25	58.80	6.86	3.56	0.30	20.21	6.84	2.8	20.21	2.80	487.27	28.00	0.1949
7	30.0	96.93	3.51	3.51	61.86	6.75	3.51	0.30	19.75	5.96	2.8	19.75	2.80	114.44	25.93	0.0424
8	17.5	92.85	19.25	1.89	55.80	0.55	19.25	0.30	28.75	24.88	3.2	28.75	9.47	43,130	55.17	29.7453
8	30.0	126.60	4.53	3.51	80.80	6.21	4.53	0.30	25.34	9.17	3.2	25.34	3.59	38,012	29.63	14.0786

**Table 9:** Results of parametric analysis.

*q*_Ed_ (kN/m^2^)	C12/15				C50/60			
	*E*_f_ = 30 GPa		*E*_f_ = 165 GPa		*E*_f_ = 30 GPa		*E*_f_ = 165 GPa	
	0.0013	0.020	0.0013	0.020	0.0013	0.020	0.0013	0.020
	*L/d*	*L/d*	*L/d*	*L/d*	*L/d*	*L/d*	*L/d*	*L/d*
5.0	22.78	22.78	22.78	22.78	35.37	34.81	35.19	34.44
6.0	20.93	20.93	20.93	20.93	33.15	32.78	33.15	32.78
7.0	19.26	19.26	19.26	19.26	30.93	30.93	30.93	30.93
8.0	17.96	17.96	17.96	18.52	28.89	28.89	28.89	28.89
9.0	17.04	17.41	17.04	17.78	27.22	27.22	27.22	27.22
10.0	16.11	16.67	16.11	17.22	25.93	25.93	25.93	25.93
11.0	15.37	16.11	15.37	16.85	24.63	24.63	24.63	24.63
12.0	14.63	15.74	14.63	16.48	23.70	23.70	23.70	23.70
13.0	14.07	15.37	14.26	16.11	22.78	22.78	22.78	23.15
14.0	13.70	15.00	13.89	15.74	21.85	21.85	21.85	22.59
15.0	13.15	14.63	13.52	15.37	21.11	21.11	21.11	22.04

**Table 10:** Slenderness limit envelope values.

*q*_Ed_	C12/15	C16/20	C20/25	C25/30	C30/37	C35/45	C40/50	C45/55	C50/60
(kN/m^2^)	*L/d*	*L/d*	*L/d*	*L/d*	*L/d*	*L/d*	*L/d*	*L/d*	*L/d*
5.0	22.78	25.00	26.85	29.07	30.74	32.41	32.96	33.70	34.44
6.0	20.93	22.78	24.44	26.67	28.15	29.44	30.93	32.04	32.78
7.0	19.26	21.11	22.59	24.63	26.11	27.41	28.52	29.81	30.93
8.0	17.96	19.63	21.11	22.96	24.26	25.56	26.67	27.78	28.89
9.0	17.04	18.52	20.00	21.67	22.96	24.07	25.19	26.30	27.22
10.0	16.11	17.59	18.89	20.56	21.67	22.78	23.89	25.00	25.93
11.0	15.37	16.85	17.96	19.63	20.74	21.85	22.78	23.70	24.63
12.0	14.63	16.11	17.22	18.70	19.81	20.93	21.85	22.78	23.70
13.0	14.07	15.37	16.67	17.96	19.07	20.00	20.93	21.85	22.78
14.0	13.70	14.81	15.93	17.41	18.33	19.26	20.19	21.11	21.85
15.0	13.15	14.26	15.37	16.85	17.78	18.70	19.44	20.37	21.11
15.0	13.15	14.26	15.37	16.85	17.78	18.70	19.44	20.37	21.11

## Data Availability

The data presented in this study are available on request from the corresponding author. The data are not publicly available due to privacy reasons.
